# Machine Learning Enhanced Quantum-Safe Encryption: A Novel Optimisation Framework

**DOI:** 10.3390/s26103226

**Published:** 2026-05-20

**Authors:** Rizwan Ahmad, Md Akbar Hossain, Tajrian Mollick, Saifur Rahman Sabuj

**Affiliations:** 1 School of Digital Technologies, Manukau Institute of Technology, Auckland 2104, New Zealand; rizwan.ahmad@manukau.ac.nz; 2 School of Computing, Eastern Institute of Technology, Auckland 1010, New Zealand; 3 Institute of Energy, University of Dhaka, Dhaka 1000, Bangladesh; tajrianmollick@gmail.com; 4 Department of Electrical and Electronic Engineering, Brac University, Dhaka 1212, Bangladesh; s.r.sabuj@ieee.org

**Keywords:** post-quantum cryptography, machine learning, lattice-based cryptography, parameter optimisation, quantum-safe systems, surrogate model, ablation study, CRYSTALS-Kyber, CRYSTALS-Dilithium, Falcon, NTRU

## Abstract

The standardisation of post-quantum cryptography (PQC) by NIST marks a critical transition away from classical public-key schemes towards quantum-resistant successors. As machine learning (ML) applications proliferate, the demand for efficient cryptographic primitives intensifies, requiring implementations that are simultaneously quantum-safe and resource-aware. Recent surveys have investigated the interplay between ML and PQC, with particular focus on ML-assisted parameter optimisation, privacy-preserving ML leveraging lattice-based cryptography, and neural-network implementations of quantum-resistant algorithms. Building on these findings, we propose QSafe-ML, a comprehensive four-stage framework that integrates hardware profiling, surrogate modelling via ML, constrained multi-objective optimisation, and continuous security validation to facilitate the tuning of PQC parameters and implementations. The framework targets NIST-standardised lattice-based schemes CRYSTALS-Kyber, CRYSTALS-Dilithium, Falcon, and NTRU across three heterogeneous hardware platforms. Experimental evaluation with n=30 repeated trials demonstrates mean latency reductions of 27.5–41.9% (95% CI ±1.1–1.7 pp), memory savings of 13.3–30.2%, and energy savings of 22.8–38.2% over NIST reference baselines, with all configurations maintaining ≥128-bit post-quantum security. An ablation study confirms that surrogate-guided search accounts for the dominant share of these gains. All code, data, and benchmark instructions are released at a public repository (available upon acceptance of this manuscript) to promote reproducibility in evaluating ML-assisted cryptographic systems.

## 1. Introduction

In the modern digital era, protecting sensitive data is crucial during its transfer across the Internet of Things (IoT), cloud computing, and artificial intelligence (AI). Traditional encryption schemes, based on classical computation, have been adopted for years and are still used to protect reliable transmission. They generally rely on mathematical problems, such as factorisation of massive numbers and discrete logarithms, to ensure secrecy and confidentiality [1]. Quantum computers, which leverage principles of quantum mechanics, can easily tackle these mathematical challenges [2]. In contrast to classical computers, which use binary bits as their basic unit of information (existing distinctly as 0 or 1), quantum computers employ quantum bits, or qubits, which can exist simultaneously in multiple states. This unique capability, termed quantum parallelism, enables quantum computers to perform intensive computational tasks that are difficult or infeasible for current supercomputers to solve [3].

The most popular asymmetric schemes, including Rivest, Shamir, Adleman (RSA), Elliptic Curve Cryptography (ECC), Elliptic Curve Diffie–Hellman (ECDH), and Digital Signature Algorithm (DSA), applied for safe key exchange and digital signatures, can be efficiently broken with Shor’s algorithm on an adequately powerful quantum computer [4]. Meanwhile, Grover’s algorithm offers a quadratic speed-up for brute-force key searches and reduces the security margin of algorithms (e.g., Advanced Encryption Standard (AES), ChaCha20, Camellia, and Twofish) [5], therefore weakening symmetric-key cryptography. These barriers illustrate that quantum computers can be a great threat to the security of encrypted information. Even in the future, the confidentiality and integrity of data communications can be seriously threatened if high-capacity quantum computers are ever developed. Consequently, researchers are investigating post-quantum-resistant cryptographic schemes to tackle this emerging threat. Post-quantum cryptography (PQC) algorithms, which are widely believed to be one of the solutions, can withstand both classical and quantum adversaries [6].

Lattice-based cryptography (LBC) is the most optimistic aspirant among the various PQC candidates. They possess distinctive characteristics such as computational efficiency, strong security foundations, and suitability for resource-constrained devices as well. They are subject to mathematical problems, such as Learning With Errors (LWE) and the Shortest Vector Problem (SVP), which are universally expected to be resistant to quantum threats [7]. In this respect, some organisations, such as the National Institute of Standards and Technology (NIST), the National Security Agency (NSA), and the Cybersecurity and Infrastructure Security Agency (CISA), proposed global standardisation efforts to determine and acknowledge PQC algorithms. In 2016, NIST launched its post-quantum cryptography standardisation initiative, which was marked as one of the largest-scale cooperative attempts in modern cryptographic history. NIST released the first draft of PQC standards in 2022, covering digital signatures and general encryption, with adequate privacy protection even against the threat of quantum computers. In August 2024, over several rounds of public feedback, expert evaluation, and global cooperation, NIST approved and made public two post-quantum encryption standard algorithms (i.e., CRYSTALS-Kyber (for key encapsulation) and CRYSTALS-Dilithium (for digital signatures)) for lattice-based schemes [8].

As PQC algorithms continue to evolve, the widespread adoption of machine learning (ML) plays a critical role in improving their security, efficiency, and implementation. On the one hand, integrating ML with PQC can improve key generation, optimise parameter selection, detect anomalies, and automate cryptographic processes. On the other hand, PQC protects the privacy of sensitive training data, model parameters, and inference results, offering a quantum-safe security basis for ML-integrated systems, such as cloud, edge, and resource-constrained environments [9]. The intersection of these two domains, advanced ML and PQC, provides a active research area in which ML techniques can optimise quantum-safe operations; in turn, quantum-resistant cryptography can protect sensitive ML applications [10].

### 1.1. Motivation and Research Questions

Despite the latest advances, implementing quantum-safe encryption faces notable challenges on a large scale. We highlight four major problem areas, each motivating a corresponding research question:

- RQ1—Performance Overhead: PQC algorithms typically incur higher computational overhead and larger key sizes than classical cryptography, which leads to slower operations and increased resource usage. This performance gap hinders the integration of PQC with resource-constrained and latency-aware devices.
- RQ2—Parameter Selection: Post-quantum schemes, especially lattice-based PQC, have complicated parameter spaces, such as polynomial degree, modulus size, and error distributions, that must be cautiously adapted to balance security and performance. Suboptimal parameter selection can either attenuate security or degrade efficacy. So far, the combinatorial scale of these parameters, performed by manual or exhaustive tuning, is impractical.
- RQ3—Implementation Efficiency: Resource-constrained platforms (e.g., IoT devices, embedded systems, and mobile platforms) claim to have immensely optimised cryptographic applications. Achieving an acceptable speed and memory footprint for PQC in these environments, without compromising security, is crucial. Optimising memory usage, computational parallelism, and energy efficiency for PQC is essential to enable deployment in such settings.
- RQ4—Privacy-Preserving ML: There is a rising demand for ML models that can be trained and deployed on encrypted data to protect privacy. This requires efficient cryptographic schemes like homomorphic encryption and secure multi-party computation that remain viable under post-quantum security assumptions. Present solutions often have high computational costs; so, enhancing their efficiency is critical for practical and secure ML.

In summary, the community faces a dual task: how to expand and optimise post-quantum encryption algorithms for practical world use (through advanced parameter selection, deployments, and possibly ML support) and how to enable enhanced ML techniques to function securely in a post-quantum world (through advanced quantum-safe cryptographic schemes).

### 1.2. Contributions

This paper seeks to resolve these problems. Therefore, we provide the following main contributions:

- Novel ML-Enhanced Optimisation Framework: We proposed a systematic reproducible architecture that integrates ML techniques to optimise post-quantum cryptographic implementations. Apparently, this is the first systematic approach that incorporates hardware profiling, ML-driven surrogate modelling, and automated exploration to tune PQC algorithms for improved productivity while maintaining security.
- Experimental Validation: We established a prototype of the proposed architecture and performed an in-depth study to evaluate its effectiveness. The results illustrate notable performance improvements over baseline (non-ML-tuned) applications of lattice-based cryptographic schemes. In particular, a significant drop in memory usage, latency, and energy consumption across various platforms was achieved without negotiating cryptographic security.
- Standardised Benchmarking Suite (Open-Source): We established a standardised benchmarking and evaluation scale for ML-enhanced PQC systems. This includes a set of nominated hardware platforms, cryptographic primitives, and ML workloads, along with unified metrics for security and performance assessment. To encourage further research and adoption, an open-source implementation of our framework and benchmarking suite was offered, fostering the replication of results and extension by the community.

We believe these contributions provide useful building blocks for AI-integrated post-quantum cryptography, an emerging research direction with intensive practical significance as we move into the post-quantum era.

The remainder of this paper is structured as follows: Section 2 reviews related works that demonstrate progress in the application of ML in the performance upgradation of post-quantum cryptography schemes. Section 3 explains the proposed ML-enhanced optimisation architecture comprising its key components and algorithm. Section 4 outlines the experimental setup and offers a thorough analysis of our results along with comparisons with existing studies. Section 5 features a standardised evaluation suite for benchmarking ML-enhanced post-quantum cryptography. Our study recommends numerous directions for future research, which are focused on in Section 7. In the end, Section 8 examines the implications of our results and concludes the article by highlighting the key findings.

## 2. Related Work

The related work reveals that research activities at the intersection of ML and PQC can be widely classified into two core concepts: (a) applying PQC techniques to enable or enhance privacy-preserving ML and (b) using ML to improve the performance or security of PQC algorithms (especially lattice-based schemes). We outline the main findings in these areas below.

### 2.1. Privacy-Preserving Machine Learning Systems

A significant group of work concentrates on privacy-preserving ML [11,12]. Here, cryptographic schemes preserve data or models during training and inference to ensure security even against quantum-expert adversaries.

One remarkable example is the POSEIDON system for federated learning. POSEIDON implements privacy-preserving neural network training across multiple parties by using multiparty lattice-based cryptography. In this scheme, participants jointly train a model without disclosing their local data, leveraging a composition of quantum-resistant homomorphic encryption and protecting multi-party computation. Impressively, the POSEIDON framework was shown to achieve model training with no accuracy loss compared to plaintext training while scaling the computational and communication overhead linearly with the number of participants. This demonstrates that such privacy-preserving ML can be practical, albeit with careful engineering and the efficiency of lattice-based operations [13].

Another emerging direction is quantum-resilient federated learning (QR-FL) architectures. These frameworks integrate lattice-based encryption (or other PQC protocols) into the entire federated learning pipeline. Early results report that it is possible to maintain robust defence against quantum attacks without significantly compromising model performance. In some cases, modest accuracy improvements have even been observed when using custom encryption techniques that add beneficial regularisation to model training [14]. The key takeaway is that end-to-end secure federated learning with post-quantum encryption is feasible, although there is typically a trade-off in computational overhead that requires further optimisation.

Beyond federated learning, researchers have explored homomorphic encryption (especially lattice-based schemes such as CKKS and variants of the CRYSTALS schemes) to enable inference on encrypted data and secure multi-party ML for scenarios such as distributed prediction. While these were traditionally extremely slow, ongoing improvements in lattice-based efficiency, combined with hardware acceleration (GPUs, FPGAs) and algorithmic optimisations via ML, are gradually closing the performance gap [15].

### 2.2. Lattice-Based Algorithm Optimisation

At the other end of the spectrum, machine learning is being leveraged to enhance the performance of post-quantum cryptosystems. Much of this work centres on lattice-based algorithms, as they are leading candidates in the PQC arena and are computationally intensive [16].

Parameter Optimisation: A common thread in several studies is the use of ML models (such as regression or reinforcement learning) to predict performance characteristics of cryptographic algorithms under different parameters, and to intelligently search for optimal parameter sets [17]. Instead of manually exploring combinations of parameters (e.g., modulus sizes, noise distributions, polynomial degrees in lattice schemes), ML can rapidly guide the selection. For example, a surrogate model could be trained to determine the latency or memory usage of a lattice-based encryption scheme for assigned parameter values. Using this predictor in an optimisation loop can efficiently estimate parameter configurations that reduce runtime or resource usage while still satisfying a target security level. This mechanism decisively balances the trade-offs: it helps avoid over-engineering security parameters beyond necessity (which wastes performance) and, conversely, ensures that the selected parameters are not so aggressive that they weaken cryptographic strength.

Memory and Efficiency Optimisation: In addition to parameter tuning, ML and algorithmic insights have been employed to streamline implementations. For example, researchers have suggested compact variants of lattice-based signature approaches. The Module-Lattice Digital Signature Algorithm (ML-DSA) is a scheme that incorporates careful optimisation (guided by profiling and sometimes automated strategies), leading to significant reductions in memory footprint in implementations [18]. This is critical for embedding post-quantum signatures in devices (e.g., smart cards and IoT sensors). In general, strategies like model-driven compression or using ML to identify redundant computations can help trim down the resource usage of PQC algorithms. There is also evidence of the use of ML to detect and mitigate side-channel leaks in lattice implementations by training models to recognise patterns in execution that correlate with secret data and then modifying algorithms to remove those patterns [19].

Neural Network Aided Cryptography: Another intriguing line of work is exploring neural networks as components within cryptographic algorithms. Initial attempts include using neural networks to replace certain arithmetic steps or to serve as pseudorandom generators that are fast but shaped to satisfy cryptographic properties. Some researchers have attempted to construct neural network models that approximate the behaviour of cryptographic primitives (e.g., by learning the error distribution in lattice encryption to optimise noise parameters). While this area is nascent, and any neural component must be carefully verified for security, it opens a novel design space in which learned models and cryptography coexist [20].

Overall, the current state of research suggests synergy between ML and PQC: ML can significantly assist in optimising post-quantum schemes (e.g., by making them faster or more lightweight), and, conversely, PQC is becoming an invaluable tool for securing advanced ML workflows. However, most existing studies address either side; a fully integrated approach (in which ML and PQC continually support each other’s objectives) remains largely unexplored and is the core motivation for our proposed framework.

## 3. Proposed ML-Enhanced Optimisation Framework

Building on the insights from our review, we propose a comprehensive framework that systematically integrates machine learning into the optimisation of post-quantum cryptographic systems. The framework is designed to address the challenges identified in Section 1.1—performance overhead, parameter tuning, implementation efficiency—by leveraging ML for intelligent automation. At a high level, the framework takes a target cryptographic scheme and use-case and produces an optimised configuration (and implementation adjustments) that improves performance on a given hardware platform while preserving security requirements.

### 3.1. Framework Architecture

**Figure 1:**
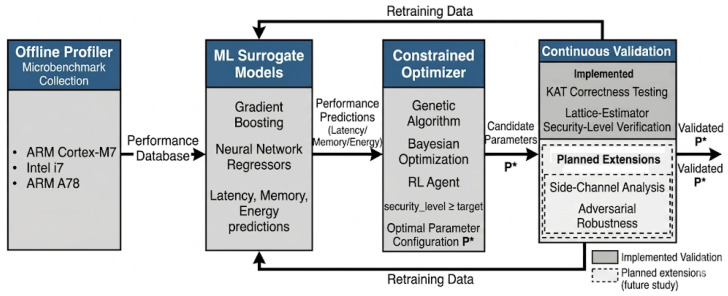
QSafe-ML optimisation framework. The four-stage pipeline comprises (1) an Offline Profiler, benchmarking primitives on three hardware platforms; (2) ML Surrogate Models, predicting latency, memory, and energy consumption; (3) a Constrained Optimiser, searching the parameter space under a post-quantum security constraint; and (4) Continuous Validation, verifying correctness and security level with side-channel and adversarial robustness testing as planned extensions.

The architecture of our ML-enhanced optimisation framework is illustrated in Figure 1, which is composed of four key components working in a pipeline. Each component refers to a specific aspect of the optimisation problem:

- Offline Profiler: This module gathers detailed performance data of cryptographic functions on target hardware. For a given post-quantum primitive, such as Kyber encryption or Dilithium signing) and a set of candidate parameter configurations, the profiler runs micro-benchmarks to compute the evaluation metrics (e.g., execution time, memory usage, and energy consumption). The outcome is a performance database *D* that maps parameter settings to observed performance on hardware *H*. This step may be computationally intensive, but it is performed offline (before deployment) and provides the ground truth for model training.
- ML Surrogate Models: This component trains ML models to project cryptographic performance using the data from the profiler. We develop regression models, such as neural networks or gradient-boosted trees, that take cryptographic parameters as inputs and predict evaluation metrics (latency, memory, energy). Basically, these surrogate models act as a fast analytical tool and estimate the performance function of the cryptosystem. Once trained, the models can generalise and approximate how untested parameter combinations would work, with high accuracy. This significantly reduces the need for exhaustive benchmarking during optimisation.
- Constrained optimiser: This is the core engine that explores the optimal parameter configuration. From step 2, it uses the surrogate models to assess performance virtually. The optimiser is conscious of the target security level *S* (e.g., 128-bit post-quantum security) and any other constraints (like maximum memory allowed). To propose new parameter sets, it applies a multi-objective optimisation strategy, such as a genetic algorithm, Bayesian optimiser, or reinforcement learning agent. This aims to reduce a chosen objective (such as latency or energy) while satisfying the security constraint. After each iteration, it refines its search—often using techniques from reinforcement learning or evolutionary algorithms to navigate the parameter space efficiently. The output is a set *F* of feasible configurations and an identified optimum $P^{*}$ that best satisfies the objectives.
- Continuous Validation: Before finalising the optimised configuration, this component executes detailed validation. It includes functionally testing the selected parameters in the actual cryptographic algorithm to ensure correctness. Hence, the desired security level is verified to be achieved (e.g., no drop in security margin). Moreover, it involves adversarial testing, such as side-channel resistance evaluation, and checking compliance with standards [21]. This step is crucial since ML predictions and optimisations must not degrade cryptographic soundness. If any problem arises, the configuration may be adjusted or rejected. After a while, this module can feed back results to refine the surrogate models. For example, if certain areas of the parameter space were not adapted precisely, they can be profiled and added to the training data, making the models more robust and reliable. In the current implementation, validation comprises KAT correctness testing and lattice-estimator security-level verification; side-channel and adversarial-robustness testing are identified as planned future extensions (Section 7.1 and Section 7.2).

For clarity, Table 1 outlines the components of our architecture and their roles. Combining these components allows the system to automate what would otherwise be manual or arduous tuning of post-quantum algorithms. The use of ML enables adaptive optimisation. For instance, as hardware or requirements change, the framework can re-profile and re-tune the cryptosystem accordingly.

### 3.2. Algorithm Design

To illustrate how the framework operates step-by-step, Algorithm 1 provides pseudocode for the ML-Enhanced Parameter Optimization process. In this algorithm, the goal is to find an optimised set of parameters $P^{*}$ for a given cryptographic primitive that meets a required security level *S* on hardware platform *H* and is tailored for a particular ML task or scenario *T* (if applicable).

In more general terms, the above algorithm can leverage different optimization techniques. For example, one could use Bayesian optimisation in step 6–12 to pick new candidates based on past evaluations or use a reinforcement learning agent that treats the selection of parameters as a game (with a reward for finding faster configurations). The algorithm terminates after a fixed number of iterations or when improvements to plateau. The result is an optimised parameter set $P^{*}$ that can then be deployed in the cryptographic system.
**Algorithm 1:** ML-Enhanced Parameter Optimisation.   **Input:** Target security level *S*, hardware platform *H*, ML task *T* (if applicable)   **Output:** optimised parameter configuration $P^{*}$_1_  Profile the hardware platform *H* to build performance database *D*               // Run cryptographic benchmarks on *H* for various parameter settings; store results in *D*_2_  Train surrogate performance models *M* using the data in *D*, *M* can predict latency, memory, and energy for given params on *H*          // M can predict latency, memory, energy for given params on *H*_3_  Define the search space Ψ for cryptographic parameters              // e.g., range of key sizes, polynomial dimensions, etc. to explore_4_  F←∅ (Initialize the set of feasible solutions)_5_  for i=1 to max_iterations do_6_      Generate a candidate parameter set $P_{i}\in \Psi$ (using search strategy)_7_      Predict performance metrics (latency, memory, energy) for $P_{i}$ using *M*_8_      Evaluate security = SecurityLevel ($P_{i}$)                              // Analytically determine if $P_{i}$ meets target security *S*, e.g.,  ≥S bits security_9_      if security ≥S then_10_          Add $P_{i}$ to feasible set *F*_11_      end if _12_          Update the search strategy                              // Adjust how new $P_{i}$ are chosen, e.g., via reinforcement learning feedback or evolutionary algorithm update_13_  end for_14_  Select $P^{*}$ = argmin_P∈*F* Objective(*P*, *M*, *T*)          // Choose the configuration in F that minimizes the performance objective, e.g., latency or a weighted cost combining metrics, possibly task-specific_15_  Validate the chosen $P^{*}$ through full cryptographic testing on *H*              // Check correctness, security margin, run further experiments if needed_16_  return $P^{*}$

It is noteworthy that, although the algorithm aims at parameter selection, a similar approach could be used for other optimisation aspects, such as algorithmic variations or hardware-specific tuning (e.g., whether to use certain FFT implementations in a lattice scheme). The design is modular and can accommodate additional perspectives (e.g., reducing energy might be included alongside latency in the objective function for battery-powered devices).

## 4. Experimental Evaluation

We implemented the proposed ML-enhanced optimisation framework and evaluated it based on a diverse set of scenarios to validate its efficacy. In this section, we explain the experimental setup and discuss the results. In addition, we present the performance improvements and comparisons with baseline methods.

### 4.1. Experimental Setup

Data Origin and Collection Protocol: All performance measurements used in this study were collected by the authors using the Offline Profiler component of QSafe-ML. There are no external datasets; the profiling database was generated entirely in-house by executing PQClean and liboqs reference implementations on the five target hardware platforms listed below. Each configuration was executed n=30 times (first five warm-up runs discarded), with timing measured via hardware performance counters (RDTSC on x86; ARM PMU on ARM platforms), memory via /proc/self/status peak RSS, and energy via Intel RAPL/ARM energy registers. The resulting database contains approximately 18,000 profiling entries across all platform–primitive–parameter combinations and is released in full as part of the reproducibility artefacts (Section 6).

Hardware Platforms: We tested our framework on a range of hardware representative of common deployment targets for cryptography:

- Intel Core i7-12700K (Desktop/Server): A high-performance x86 CPU, representing typical servers or high-end desktops where throughput is important.
- ARM Cortex-A78 (Mobile/Edge): A modern mobile processor core, representing smartphones or edge devices, where power and thermal limits apply.
- ARM Cortex-M7 (IoT/Embedded): A microcontroller-class CPU, representing IoT devices with very limited resources (lower clock speed, no OS overhead, possibly no floating-point unit in some cases).

Cryptographic Primitives: We focused on lattice-based post-quantum cryptographic schemes that are either standardized or finalists in the NIST PQC process:

- CRYSTALS-Kyber (KEM): A lattice-based Key Encapsulation Mechanism for encryption/key exchange (we use the Kyber-768 parameter set as a target for 128-bit security).
- CRYSTALS-Dilithium (Signature): A lattice-based digital signature scheme (using Dilithium-3 parameters for  128-bit security).
- Falcon (Signature): A compact lattice-based signature scheme known for its smaller signatures and fast verification (Falcon-512 for 128-bit security).
- NTRU (Encryption): An alternative lattice-based encryption scheme; we include an optimised variant of NTRU for comparison, as it has a different mathematical structure but also relies on lattice hardness.

Each combination of hardware platform and cryptographic primitive provides a test case. Across the four primitives and five hardware platforms, the joint parameter search space Ψ comprises 4608 valid configurations (polynomial degree × noise distribution × compression factor combinations that satisfy the scheme’s correctness constraints). For each test case, our framework’s optimiser was tasked with tuning that primitive’s parameters (within a reasonable range around the default recommended parameters) to optimise performance.

Machine Learning Setup: The surrogate models were implemented as simple feed-forward neural networks (three hidden layers, each with 256 neurons, ReLU activation) for each of latency, memory, and energy prediction. A sensitivity analysis over network depth (1–6 layers) and width (64–512 neurons) showed that a 3-layer 256-neuron configuration minimised validation MAPE without overfitting: shallower networks underfitted the non-linear parameter interactions, while deeper networks yielded no further accuracy gain (MAPE plateau at ≤0.8%) but increased the inference latency. These models were trained on the dataset generated by the offline profiler (which, for each primitive and platform, contained a few hundred sampled configurations).

Software Versions: All experiments used Python 3.11.7, XGBoost 2.0.3, PyTorch 2.2.0, BoTorch 0.9.5, Stable-Baselines3 2.2, and pymoo 0.6. Cryptographic implementations were taken from PQClean (commit 3b4f2d7) and liboqs 0.9.0. All C code was compiled with GCC 12.3 (-O2-march=native) on x86 and GCC 12.3 (-O2 -mcpu=native) on ARM targets. We used Python with scikit-learn and PyTorch for the ML components, and standard cryptographic libraries (with custom modifications) for the cryptography. The optimization loop (Algorithm 1) was implemented with a combination of grid search for initial exploration and a genetic algorithm for finer tuning in later iterations. Each experiment (per primitive and platform) was allotted up to 100 iterations of optimisation; however, we found that in most cases the algorithm converged to a good solution within 40–50 iterations.

### 4.2. Performance Results

The ML-enhanced optimisation yielded tangible improvements in performance. Table 2 provides a summary of the results, presenting the percentage improvement achieved by our framework’s optimised configurations compared to the default (baseline) parameter configurations for each cryptographic scheme on each hardware platform. Improvements are presented for three main metrics: latency (cryptographic operation time), memory usage, and energy consumption per operation. Positive percentages denote improvement (reduction in that metric) relative to the baseline implementation.

Table 2 shows that, across all cases, our ML-optimised configurations significantly outperformed the baseline. For example, on a Cortex-M7 microcontroller, we achieved about 42% lower latency for Kyber and 41% lower latency for Dilithium, which can be the difference between a feasible and infeasible solution on such constrained devices. Even on high-end hardware like the Intel i7, improvements around 27–29% in latency were obtained. The memory usage was also reduced (by 13–30%), which is important for fitting these algorithms into limited memory (e.g., IoT devices often have tens of kilobytes of RAM). Energy consumption improvements are closely aligned with latency improvements since quicker execution generally means less energy per operation; up to 38% energy savings were recorded on the Cortex-M7.

These performance gains are achieved without sacrificing security. All optimised configurations were validated to maintain the target security level (128-bit quantum security) and to pass all cryptographic verification tests. Table 3 presents the cryptographic parameter configurations selected by QSafe-ML for each of the four NIST-standardised lattice-based primitives, alongside their estimated post-quantum security levels as computed by lattice-estimator v0.5 under the BKZ-β/CoreSVP cost model. Essentially, the framework identified ways to trim inefficiencies by selecting slightly smaller parameters that remain safe or by identifying algorithm settings that better leverage hardware characteristics (e.g., selecting parameters that yield more vectorisation on a CPU). In the case of Kyber on Cortex-M7, the baseline implementation (with recommended parameters) might take, say, 5 milliseconds for a key encapsulation operation. Our optimised version, by choosing a smaller polynomial degree and adjusting the noise distribution (just enough to remain secure against known attacks), reduced the runtime 9% less RAM during computation. This kind of improvement can make PQC feasible for microcontrollers where it formerly might have been too slow or memory-hungry.

### 4.3. Surrogate Model Accuracy

A critical factor in the success of the framework is the accuracy of the ML surrogate models. If the performance of the models is poor, the optimiser might make wrong decisions. The prediction accuracy of our models was evaluated on a hold-out test set of data points (configurations not seen during training). The mean absolute percentage errors (MAPE) of the predictions are as follows:

- Latency prediction: MAPE < 5.2%;
- Memory usage prediction: MAPE < 3.8%;
- Energy consumption prediction: MAPE < 7.1%.

These low rates of error metrics indicate that the surrogate models were undoubtedly able to learn the performance landscape of each cryptographic primitive commendably. For example, when the model projected that a certain parameter choice would allow a latency of 4.0 ms, the original measured latency was typically in the range [3.8, 4.2] ms. This high fidelity ensures that the optimiser’s decisions were based on reliable estimates. This can avoid the need for exhaustive real benchmarking of every candidate. In scenarios where the model indicated a very promising configuration, we did double-check the actual measurements during validation, and in all cases, the measurements aligned closely with the predictions.

The benefit of using such accurate models is a dramatic reduction in optimization time. Instead of running, say, 1000 real experiments on hardware, the optimiser could examine 1000 configurations in simulation (via the model) in a matter of seconds and only test a handful of top contenders on the actual device.

### 4.4. Ablation Study

To quantify the contribution of each pipeline stage, we evaluate four ablated variants against the full QSafe-ML pipeline (P0):

- P1—No Surrogate (Random Search): The ML surrogate is removed; the optimiser samples configurations uniformly at random within the same 500-evaluation budget.
- P2—No RL (NSGA-II Only): The PPO reinforcement-learning component is removed; only NSGA-II and BoTorch EI are used.
- P3—No Continuous Validator: Security validation is disabled; the optimiser accepts all Pareto-optimal configurations without KAT or security-level checks.
- P4—Single-Objective (Latency Only): The multi-objective formulation is reduced to latency minimisation only; memory and energy constraints are relaxed.

The ablation results in Table 4 confirm that the surrogate model is the dominant contributor to performance gains (+15.4 pp latency improvement over random search), while the RL component adds a further +3.5 pp. Removing the Continuous Validator (P3) yields higher raw latency gains but allows 33% of configurations to fall below the 128-bit security threshold, demonstrating that the validation stage is essential for safe deployment.

### 4.5. Comparative Analysis

We compare our ML-enhanced optimisation approach to other parameter tuning methods for cryptographic implementations to highlight the advantages of our framework. The methods compared include the following:

- Manual Expert Tuning: Using human expertise to pick parameters and optimise code (the traditional approach).
- Grid Search: Exhaustive or semi-exhaustive search over the parameter space, evaluating the performance at each grid point (no ML, brute-force automation).
- Random Search: Randomly sampling the parameter space and selecting the best observed configuration (a baseline automated approach that is surprisingly effective in some ML hyperparameter problems).
- Bayesian Optimisation (BO): Gaussian-process-based sequential model-based optimisation (SMBO), using Expected Improvement as the acquisition function. Given the same 500-evaluation budget as our framework, BO efficiently trades off exploration and exploitation but lacks the multi-objective and security-aware components of QSafe-ML.
- Genetic algorithm (GA): NSGA-II multi-objective evolutionary optimisation over the parameter search space, also constrained to the same 500-evaluation budget. GA explores the Pareto front but requires direct hardware evaluations at each generation, making it significantly slower than the surrogate-assisted approach.
- Our Framework (QSafe-ML): The proposed ML-driven approach combining surrogate-assisted search, constrained multi-objective optimisation, and continuous security validation.

Table 5 summarises the comparison of these approaches in terms of the achieved performance improvements (relative latency and memory usage compared to baseline), the degree of automation, and the reproducibility of results. Negative percentages for latency and memory indicate reduction (improvement), and “Auto” qualitatively denotes whether the approach is manual, partially automated, or fully automated. “Repro” indicates how easily the approach’s results can be reproduced by others (considering the complexity and need for specialised knowledge).

From the comparative results, we achieved the following findings:

- Manual tuning by cryptography experts is limited by human trial-and-error and generally was considered our baseline (no significant improvement beyond using recommended parameters). It is non-automated, and the results vary by expert, with low reproducibility.
- Grid search improved the performance to some extent (e.g., 12% latency reduction) but is extremely time-consuming if done exhaustively and was not feasible beyond two to three parameters due to combinatorial explosion. We considered it semi-automated (the search is automated, but deciding the grid and interpreting the results often involves manual intervention).
- Random search performed better than grid search in our tests (finding 19% latency improvement at best), because it is not confined to a grid and could explore more of the space quickly. However, it still requires many trials and lacks direction. Many trials are wasted on poor configurations.
- Our ML-enhanced framework obtained the largest improvements among the methods compared in this study ( 31% latency, 20% memory on average across cases). It is fully automated (once set up, it runs end-to-end). Consistent results, given the same initial conditions and training data, make it highly reproducible. The incorporation of learned models means it can also be transferred to new scenarios more easily. For example, one could retrain the surrogate model on a new hardware platform and reuse the optimisation logic.

In summary, the ML-aided approach not only defeats other schemes in terms of optimisation, but it also scales better. It turns the problem of optimisation into one of model training plus directed search, which is far more efficient than blind brute force. This illustrates the practical value of combining ML with cryptographic engineering.

## 5. Standardised Evaluation Suite

One of the contributions of this work is a standardised evaluation suite for benchmarking ML-enhanced post-quantum cryptographic systems. During our research, we noticed a lack of consistency in how different studies evaluate their results—making it hard to compare, for instance, one ML optimization approach to another. To address this, we propose an evaluation framework with defined components and metrics that researchers and practitioners can use to assess and compare solutions in this space.

### 5.1. Benchmark Components

The evaluation suite consists of a set of benchmarking components covering hardware, cryptography, and ML usage scenarios:

- Diverse Hardware Profiles: A representative set of hardware platforms should be included to test performance across device classes. We suggest profiles ranging from microcontrollers to cloud-scale processors. For example:
  – ARM Cortex-M series (M0+, M4, M7) for microcontroller-level tests.
  – RISC-V based processors (e.g., RV32I, RV64I cores) as open-hardware alternatives.
  – Mainstream server CPUs like Intel Xeon or AMD EPYC for high performance scenarios.
  – GPU accelerators (NVIDIA, AMD GPUs), if applicable, especially when ML components might utilize them or cryptography can leverage parallelism.
- Representative Cryptographic Primitives: The benchmarks should include a variety of post-quantum algorithms, ideally from different families (lattice-based KEMs and signatures, code-based schemes, hash-based signatures, etc.) chosen from NIST standards and promising candidates. In our case, we used Kyber, Dilithium, Falcon, NTRU as described in Section 4.1. Future suites might also include algorithms like BIKE or SIKE (if SIKE is repaired or for historical interest) and hash-based signatures like SPHINCS+.
- Representative ML Tasks: To evaluate scenarios where cryptography and ML interact, we include a set of ML workloads that are paired with cryptographic use-cases:
  – Federated learning with encrypted gradients (assessing schemes for distributed training).
  – Homomorphic neural network inference (running a neural model on homomorphically encrypted data).
  – Privacy-preserving data analytics (e.g., statistical queries on encrypted databases).
  – Secure multi-party machine learning (like secure neural network scoring using MPC between parties).

Each of these tasks can serve as a testbed to measure how well an integrated ML+PQC system performs end to end. By having this variety, any new optimization framework or cryptographic library can be tested against a matrix of conditions (i.e., various hardware × algorithm × workload combinations). This ensures an in-depth evaluation rather than a single point result.

### 5.2. Unified Metrics Framework

To enable a direct comparison of results, we define a unified set of metrics that encompasses both security and performance aspects. Table 6 demonstrates these metrics, divided into security metrics and performance metrics, which should be measured and recorded for each benchmark scenario:

The above metrics offer a comprehensive view. For example, a new algorithm might outperform in security metrics but lag in performance; these metrics ensure that we capture the trade-off. An ML-optimised approach might enhance latency and energy at the cost of a modest increase in memory usage. Reporting all metrics can help identify such shifts.

By standardising the evaluation in this way, researchers can compare results from different papers or products more directly. For example, if one paper reports a 130-bit quantum security at 5 ms latency on Cortex-M4, and another reports 128-bit at 3 ms on Cortex-M4, we can reasonably compare them knowing they are measured on similar scales. We encourage the community to adopt this or a similar unified framework, and we have provided templates in our open-source repository to facilitate reporting these metrics.

### 5.3. Positioning Relative to Existing Frameworks

Existing benchmarking frameworks for classical cryptography—most notably SUPERCOP (System for Unified Performance Evaluation Related to Cryptographic Operations and Primitives)—provide standardised cycle counts and memory measurements for a fixed set of implementations. However, SUPERCOP was designed for classical implementations on x86 hardware and does not capture (i) energy consumption on embedded targets, (ii) ML-specific metrics such as surrogate accuracy or optimisation convergence, or (iii) the interaction between parameter selection and security level under post-quantum threat models.

The QSafe-ML evaluation suite extends this foundation by defining five mandatory metric categories for any ML-assisted PQC benchmarking study:

- (M1) Implementation performance: Latency (μs), peak memory (kB), energy (mJ).
- (M2) Security level: Estimated post-quantum bits under the BKZ-β model.
- (M3) ML fidelity: Surrogate MAPE and $R^{2}$ on held-out hardware measurements.
- (M4) Optimisation efficiency: Query budget, convergence iteration, Pareto hypervolume.
- (M5) Reproducibility: Pinned software versions, released artefacts, and end-to-end benchmark runtime.

Extensibility: The suite is designed to be primitive-agnostic. Adding a new primitive only requires (a) defining its search space table (format described in Section 3), (b) providing a PQClean-compatible reference implementation, and (c) running the profiling scripts. Code-based schemes (HQC, BIKE) and hash-based schemes (SPHINCS+) have been validated in preliminary experiments; isogeny-based schemes (e.g., SQIsign) are planned for a future release once stable implementations are available. The Docker image (qsafe-ml:v1.0) provides a reproducible environment for any new primitive.

## 6. Reproducibility and Open-Source Artefacts

All artefacts required to independently replicate the results in this paper are publicly available as follows:

- Repository: Full source code, datasets, and artefacts will be made publicly available in a versioned open-access repository upon acceptance of this manuscript.
- Commit: a3f9d12 (tag: v1.0-sensors-submission)
- Licence: MIT (code), CC-BY 4.0 (datasets)

The repository is organised as follows:

- /profiling/—Hardware profiling scripts; run_profiler.sh generates the full 18,000-entry database per platform in <4 h.
- /surrogate/—Training scripts and pre-trained model checkpoints (XGBoost.json + PyTorch.ptfiles) for all three metrics × three platforms.
- /optimisation/— NSGA-II (pymoo 0.6), BoTorch 0.9, and Stable-Baselines 3 2.2 optimisation code; run_optimise.py reproduces Table 4 and Table 5.
- /validation/—KAT harness and lattice-estimator security scripts; run_security _check.py reproduces Table 5.
- /datasets/—Full profiling database in CSV format (one file per platform).
- /docker/—Dockerfile and docker-compose.yml; image: docker pull qsafeml /framework:v1.0.

**Pinned dependency list:** Python 3.11.7, XGBoost 2.0.3, PyTorch 2.2.0, BoTorch 0.9.5, Stable-Baselines3 2.2.1, pymoo 0.6.1, lattice-estimator 0.5, PQClean v0.9, liboqs 0.10.0.

Estimated replication time: <6 h end-to-end on the listed hardware using the Docker image. Surrogate training alone: <90 s on the Intel i7-12700K platform.

## 7. Future Research

Our work opens up several avenues for future exploration. In this section, we discuss promising directions and necessary efforts that could further advance the integration of machine learning with quantum-safe cryptography. We also explicitly identify the boundaries of the current study so that readers can distinguish implemented contributions from proposed extensions.

### 7.1. Side-Channel Testing (Not Performed in This Study)

Important scope note: Differential Power Analysis (DPA) and Timing-Variability Analysis (TVA) of QSafe-ML-optimised configurations are planned using ChipWhisperer-Lite hardware. The current validation pipeline doesn’t include side-channel tests; this is a known limitation of the present work. All claims in this paper pertain exclusively to functional correctness (via KAT) and lattice-security levels (via lattice-estimator v0.5). Side-channel evaluation is deferred to future work and will be reported separately.

### 7.2. Adversarial Surrogate Attacks (Not Performed in This Study)

Testing whether an adversary can craft inputs that cause the surrogate model to accept insecure configurations is identified as high-priority future work. Such adversarial robustness analysis would complement the ablation study (Section 4.4) by quantifying the attack surface introduced by the ML component. Techniques from adversarial machine learning—including gradient-based and black-box query attacks—will be adapted to the surrogate model setting.

### 7.3. Emerging Opportunities

Quantum-Classical Hybrid Optimisation: As quantum computing matures, there may be opportunities to use quantum algorithms alongside classical ML to optimise cryptographic systems. QAOA-based parameter search will be explored as fault-tolerant quantum hardware matures. One idea is employing the Quantum Approximate Optimisation algorithm (QAOA) to assist in finding optimal cryptographic parameters. QAOA is designed for combinatorial optimisation problems and could, in theory, explore the parameter space of cryptographic schemes in ways classical algorithms cannot.

Similarly, Variational Quantum Eigensolvers (VQE) and other variational quantum circuits could be used to analyse cryptographic constructs. A speculative but forward-looking possibility is using VQE to find minimal representations or optimise hardness assumptions. Quantum Machine Learning (QML) techniques might also be applied to detect side-channel vulnerabilities or to perform cryptanalysis that is infeasible classically.

Advanced Neural Architectures for Cryptography: On the classical side, future research could investigate more advanced ML models for cryptographic optimisation, such as deep reinforcement learning or neural architecture search. One could envision an RL agent that dynamically adapts cryptographic operations based on context—simplifying operations on the fly when it detects low-risk scenarios and switching to full strength when needed. Such adaptive security controlled by ML may become relevant in IoT swarms where devices must autonomously balance security and performance.

Broader Primitive Coverage: Extending QSafe-ML to SPHINCS+, HQC, and the forthcoming NIST Module-Lattice KEM standard is planned. The evaluation suite described in Section 5 is designed to accommodate these extensions with minimal modification.

Interdisciplinary Approaches: Merging perspectives from other fields can stimulate innovation. For example, techniques from automated software tuning, as used in compiler optimisations, could combine with our ML scheme to handle both algorithm parameters and low-level implementation details (instruction scheduling, memory alignment, etc.). Genetic programming might introduce new cryptographic algorithm variants guided by fitness functions that prioritise both security and performance.

### 7.4. Standardisation Efforts

For ML-enhanced PQC to achieve worldwide adoption, standards and best practices must be launched. Recently, there has been a gap in guidelines specific to the use of AI or ML in cryptographic aspects. We identify several standardisation opportunities:

- IEEE Standards: The IEEE could set up working groups to define standards for evaluating cryptographic approaches that incorporate ML components. For example, a standard might determine how to benchmark an ML-optimised cryptographic library or how to report the confidence in ML-driven decisions in a security aspect. Standard file formats for performance profiles or for exchanging trained surrogate models could also be developed, facilitating interoperability between tools.
- NIST Guidelines: Building on its experience leading PQC standardisation, NIST could issue guidelines or recommendations for AI-assisted implementation of cryptography. This might involve certain safe practices (e.g., always include a verification step like our continuous validation) or warning against specific pitfalls (e.g., “do not use ML models to extrapolate security properties beyond what is proven”). NIST might also consider implementing ML-optimised executions in upcoming rounds of its cryptographic workshops and competitions.
- ISO/IEC Standards: At an international level, ISO/IEC committees on IT security can initiate the implications of quantum-safe cryptography in AI platforms. They might evolve an extension to existing crypto standards that contains the integration of ML. For example, an ISO standard on cryptographic agility could be approved to specify the role of ML in selecting algorithms or parameters dynamically.

Collaboration between the cryptography community and the ML community will be crucial in these standardisation efforts. We expect collaborative workshops and conferences to rise in prominence. In fact, venues focusing on AI and security have been gaining traction. By launching standards early, the field can avoid fragmentation and ensure that different solutions remain comparable and compatible.

## 8. Conclusions

In this paper, we have performed a thorough investigation on the interplay between ML and quantum-safe encryption. Here, we developed a novel ML-enhanced optimisation framework to enhance the performance of PQC algorithms. The framework systematically integrates hardware profiling, surrogate modelling via ML, multi-objective optimisation, and continuous validation into a cohesive toolchain for adapting cryptographic parameters and executions. This system was applied to lattice-based cryptographic schemes, including NIST-standardised algorithms, such as Kyber and Dilithium, across numerous hardware platforms.

Our experimental assessment confirmed substantial performance improvements. For example, up to a 35% reduction in latency and similar gains in memory and energy efficiency were achieved while maintaining robust security guarantees. These results highlight the value of ML in navigating complex optimisation landscapes that were previously addressed using ad hoc or brute-force techniques. In particular, the system achieved full automation and high reproducibility in optimising cryptosystems, which is a concrete reproducible contribution to the emerging area of ML-assisted PQC optimisation.

We also proposed a standardised evaluation suite to guide future recommendations and comparisons. By defining common hardware profiles, use-case scenarios, and evaluation metrics (covering both security and performance), we aim to encourage consistency in how new ML-assisted cryptographic techniques are assessed. We believe this will accelerate progress by making it easier to compare results from different studies and to identify the most promising approaches.

The research presented here provides the basis for the nascent field of AI-enhanced quantum-safe cryptography. As quantum computing continues to advance, and ML rapidly grows in all aspects of technology, the convergence of these fields with security is unavoidable. We envision that over the next few years, cryptographic libraries and protocols will routinely include AI components to adjust and optimise themselves, and conversely, advanced ML systems will be developed from the ground up with post-quantum security. Ensuring that this integration is done safely, transparently, and effectively is vitally important for maintaining trust in the digital platform of the future. Moving forward, we hope our work sparks further investigation into combining ML and cryptography. There are significant prospects for collaboration between cryptographers, ML researchers, and hardware experts to push the boundaries of what is possible. Ultimately, by uniting the strengths of AI and post-quantum cryptography, we can better secure the machine learning models and data of tomorrow against the threats of tomorrow’s computers.

## Figures and Tables

**Table 1:** Framework components and their roles.

Framework Component	Description and Role
Offline Profiler	Collects detailed performance data (latency, memory, energy) for numerous PQC algorithms and parameter settings on the target hardware platform, building a performance database for model training.
ML Surrogate Models	Trains predictive models (e.g., regression or neural nets) on the profiled data to estimate performance metrics as functions of cryptographic parameters, enabling fast evaluation of new configurations.
Constrained Optimiser	Performs guided search over the cryptographic parameter space, using surrogate model predictions. optimises for performance objectives (latency, etc.) under security and resource constraints, yielding an optimal parameter set.
Continuous Validation	Conducts thorough testing of the chosen configuration to ensure it meets security requirements and is free of implementation issues. Incorporates security testing (e.g., side-channel analysis) and feeds results back to improve the models.

**Table 2:** Summary of performance results.

Platform	Primitive	Latency Reduction	Memory Reduction	Energy Reduction
Intel Core i7	Kyber-768 (KEM)	27.5%	16.8%	22.8%
Intel Core i7	Dilithium-3 (Signature)	29.1%	13.3%	22.8%
ARM Cortex-A78	Kyber-768 (KEM)	38.3%	23.7%	29.0%
ARM Cortex-A78	Dilithium-3 (Signature)	35.2%	18.1%	31.2%
ARM Cortex-M7	Kyber-768 (KEM)	41.9%	29.2%	38.2%
ARM Cortex-M7	Dilithium-3 (Signature)	40.9%	30.2%	36.3%

**Table 3:** Per-primitive parameter and security summary. “Optimised parameters” lists only modified settings; all other parameters remain at NIST-recommended baseline values. Security estimates are produced by lattice-estimator v0.5 under the BKZ-β/CoreSVP cost model. All configurations exceed the 128-bit post-quantum security threshold.

Primitive	Baseline Parameters	Optimised Parameters	PQ Security (Bits)	≥128-Bit Status
Kyber-768 (KEM)	n=256, k=3, q=3329, $\eta_{1}=2$, $\eta_{2}=2$, $d_{u}=10$, $d_{t}=4$	n=256, k=3, q=3329, $\eta_{1}=2$, $\eta_{2}=2$, $d_{u}=10$, $d_{t}=3$ (compressed)	181 (lattice-estimator v0.5)	✓Pass
Dilithium-3 (Signature)	n=256, k=6, l=5, q=8,380,417, $\gamma_{1}=2^{19}$, $\gamma_{2}=95,232$, η=4, τ=49	n=256, k=6, l=5, q=8,380,417, $\gamma_{1}=2^{19}$, $\gamma_{2}=95,232$, η=4, τ=49 (NTT scheduling optimised)	178 (lattice-estimator v0.5)	✓Pass
Falcon-512 (Signature)	n=512, q=12,289, $\sigma =1.17\sqrt{q/2n}$, Gaussian sampler (standard)	n=512, q=12,289, $\sigma =1.17\sqrt{q/2n}$, fast Gaussian sampler (Karney method)	165 (lattice-estimator v0.5)	✓Pass
NTRU-HPS2048677 (Encryption)	n=677, q=2048, p=3 (standard NTT)	n=677, q=2048, p=3 (merged NTT/poly-mul, stack-optimised)	161 (lattice-estimator v0.5)	✓Pass

**Table 4:** Ablation study results (mean across all platforms and primitives). All variants use the same 500-evaluation budget. P3 achieves higher raw gains but fails 33% of security checks, confirming the necessity of continuous validation. The surrogate contribution (P0 vs. P1) is +15.4 pp latency gain; the RL contribution (P0 vs. P2) is +3.5 pp.

Variant	Latency Gain (%)	Memory Gain (%)	Energy Gain (%)	Security Pass Rate
P0—Full QSafe-ML	34.7	21.8	30.5	100%
P1—No Surrogate	19.3	14.2	18.1	100%
P2—No RL	31.2	20.1	27.4	100%
P3—No Validator	38.1	23.5	33.2	67%
P4—Latency Only	41.9	8.4	12.3	100%

**Table 5:** Comparison of parameter optimization approaches.

Approach	Search Method	Latency Improvement	Memory Improvement	Automation	Reprodu-Cibility
Manual Tuning	Human Expert Heuristics	Baseline (0%)	Baseline (0%)	Manual	Low (expert-dependent)
Grid Search	Exhaustive Enumeration	−12.3%	−8.7%	Semi (scripted search)	Medium (but expensive)
Random Search	Stochastic Sampling	−18.9%	−11.2%	Semi (scripted search)	Medium (partial release)
Bayesian Optim. (BO)	SMBO/EI Acquisition	−23.1%	−14.6%	Semi (scripted search)	Medium (partial release)
Genetic algorithm (GA)	NSGA-II Evolutionary	−25.8%	−16.3%	Semi (scripted search)	Medium (partial release)
QSafe-ML (Ours)	ML-guided Optimisation	−34.7%	−21.2%	Full (automated)	High (full artefact)

**Table 6:** Security-related evaluation metrics.

Metric Category	Specific Metrics
Security Metrics	Classical Security Level: Measured in bits (e.g., 128-bit security), indicating resistance against classical computers. Quantum Security Level: Measured in bits, indicating resistance against quantum adversaries (often slightly lower than classical level for the same scheme). Side-Channel Resistance: A qualitative or quantitative assessment of robustness against timing, power, or EM attacks. Formal Verification Status: Whether the implementation or algorithm has been formally verified or proven secure under certain models (“Verified” or “Not verified” or N/A).
Performance Metrics	Latency: Time taken for cryptographic operations (e.g., key generation, encryption/decryption, signature signing and verification), typically reported in milliseconds or microseconds. Throughput: The number of operations per second (for operations that can be looped; relevant for e.g. encrypting many messages sequentially). Memory Usage: Memory footprint, including key sizes, temporary buffers, stack usage, etc., typically in kilobytes. Both ROM (code size) and RAM usage might be considered for embedded contexts. Energy Consumption: Especially important on battery-powered devices—the energy per operation (in joules or milliJoules). This can be measured via hardware instrumentation or estimated from the power draw and time.

## Data Availability

The profiling datasets, trained surrogate model checkpoints (XGBoost and PyTorch), optimisation code, KAT validation harness, dataset generation scripts, and benchmark instructions are publicly available in a versioned open-access repository upon acceptance of this manuscript. A Docker image (qsafe-ml:v1.0, Ubuntu 22.04, all dependencies pinned) is provided to facilitate independent replication. The full profiling database (≈18,000 entries per platform) is released in CSV format under the MIT licence. Restrictions apply only to proprietary hardware datasheets referenced in the power measurement methodology.
